# Exploring monovalent and multivalent peptides for the inhibition of FBP21-tWW

**DOI:** 10.3762/bjoc.11.80

**Published:** 2015-05-11

**Authors:** Lisa Maria Henning, Sumati Bhatia, Miriam Bertazzon, Michaela Marczynke, Oliver Seitz, Rudolf Volkmer, Rainer Haag, Christian Freund

**Affiliations:** 1 Institute for Chemistry and Biochemistry, Protein Biochemistry Group, Thielallee 63, Freie Universität Berlin, 14195 Berlin, Germany; 2 Institute for Chemistry and Biochemistry, Freie Universität Berlin, Takustr. 3, 14195 Berlin, Germany; 3 Institute for Chemistry, Humboldt-Universität Berlin, Brook-Taylor-Str. 2, 12489 Berlin, Germany; 4 Leibniz Institut für Molekulare Pharmakologie FMP, Robert-Rössle-Str.10, 13125 Berlin, Germany; 5 Institute of Medical Immunology, Charité-Universitätsmedizin Berlin, Berlin, Germany

**Keywords:** FBP21-tWW, isothermal titration calorimetry, multivalent polymers, polyglycerol peptide conjugates, proline-rich sequence recognition

## Abstract

The coupling of peptides to polyglycerol carriers represents an important route towards the multivalent display of protein ligands. In particular, the inhibition of low affinity intracellular protein–protein interactions can be addressed by this design. We have applied this strategy to develop binding partners for FBP21, a protein which is important for the splicing of pre-mRNA in the nucleus of eukaryotic cells. Firstly, by using phage display the optimized sequence WPPPPRVPR was derived which binds with *K*_D_s of 80 μM and 150 µM to the individual WW domains and with a *K*_D_ of 150 μM to the tandem-WW1–WW2 construct. Secondly, this sequence was coupled to a hyperbranched polyglycerol (hPG) that allowed for the multivalent display on the surface of the dendritic polymer. This novel multifunctional hPG-peptide conjugate displayed a *K*_D_ of 17.6 µM which demonstrates that the new carrier provides a venue for the future inhibition of proline-rich sequence recognition by FBP21 during assembly of the spliceosome.

## Introduction

Pre-mRNA splicing is an important step in the expression of eukaryotic genes, during which non-coding elements are removed from the pre-mRNA and coding elements are ligated to form a mRNA which can further on be translated into protein. The use of alternative splice sites represents a means to enhance the post-transcriptional diversity of transcripts and ultimately of the proteome of eukaryotic species. Alternative splicing is rare in yeast but a commonality in higher eukaryotes, for which the existence of different splice isoforms is the rule rather than the exception [1]. Aberrant splicing is associated with several diseases [2] and the inhibition of splicing factors has become a recent topic in the field of antitumor drugs [3]. Formin-binding protein 21 (FBP21) has been detected as a component of early spliceosomal complexes and more specifically was shown to interact with proline–arginine-rich peptides in the core splicing protein SmB/B’ and the U2-associated protein SF3B4. The interaction of FBP21 with these proteins is conferred by two WW domains that are connected by a short, 8 amino acid long linker sequence. Multivalent recognition of the proline-rich sequences (PRS) by the tandem-WW domains was shown to boost overall affinity, while still keeping the interaction highly dynamic [4–5]. FBP21 was shown to enhance splicing of a reporter construct in living cells [4] while another study suggested that the protein is involved in the alternative splicing of vascular endothelial growth factor (VEGF). In the same study, the natural compound borrelidin was suggested to confer its splicing inhibition function by directly binding to the WW domains of FBP21 [6]. Here, we have taken a different approach to inhibit binding of FBP21-tWW to proline-rich sequences in the spliceosome, where the optimization of a peptide ligand by phage screening and subsequent multivalent display on a dendritic polymer is combined to create higher affinity binders with the potential to be used in cellular studies.

Dendritic polyamines such as polyglycerol amine [7–8], polyethyleneimine [9–10], and polyamidoamine [11] are taken up by the cell and localize to endosomes or endolysosomes, where they lead to proton pumping and concomitant influx due to a proton sponge effect [12], increasing the ionic strength in these organelles. Eventually, this leads to osmotic rupture of the endosome and release of the dendritic polymer into the cytoplasm [13]. These polymeric scaffolds have been explored well for tumor targeting by using polymer-drug conjugates or polyplexes with genes or siRNA [14], but also have the potential to inhibit protein–protein interaction in cells, by displaying multiple ligands for a target protein. The hyperbranched polyglycerol amine (hPG-NH_2_) with different degrees of amine functionalization can easily be prepared from hPG-OH with high yields in three steps, as reported in the literature [15]. It can be used for peptide coupling, while it is still maintaining the minimal positive charge on the carrier polymeric backbone which is necessary for cell penetration. The hPG-NH_2_ **1** with 70% amine functionalization was chosen to conjugate multiple copies of an optimized targeting peptide, yielding the multivalent hPG-peptide conjugate **2**. The dissociation constant (*K*_D_) of the interaction between hPG-peptide conjugate **2** and FBP21-tWW was measured by isothermal titration calorimetry (ITC) and compared to the *K*_D_ of the interaction between the monovalent peptide and FBP21-tWW to analyze if multivalent display in form of the hPG-peptide conjugate **2** increases binding affinity.

## Results and Discussion

### Phage display

**Figure 1:**
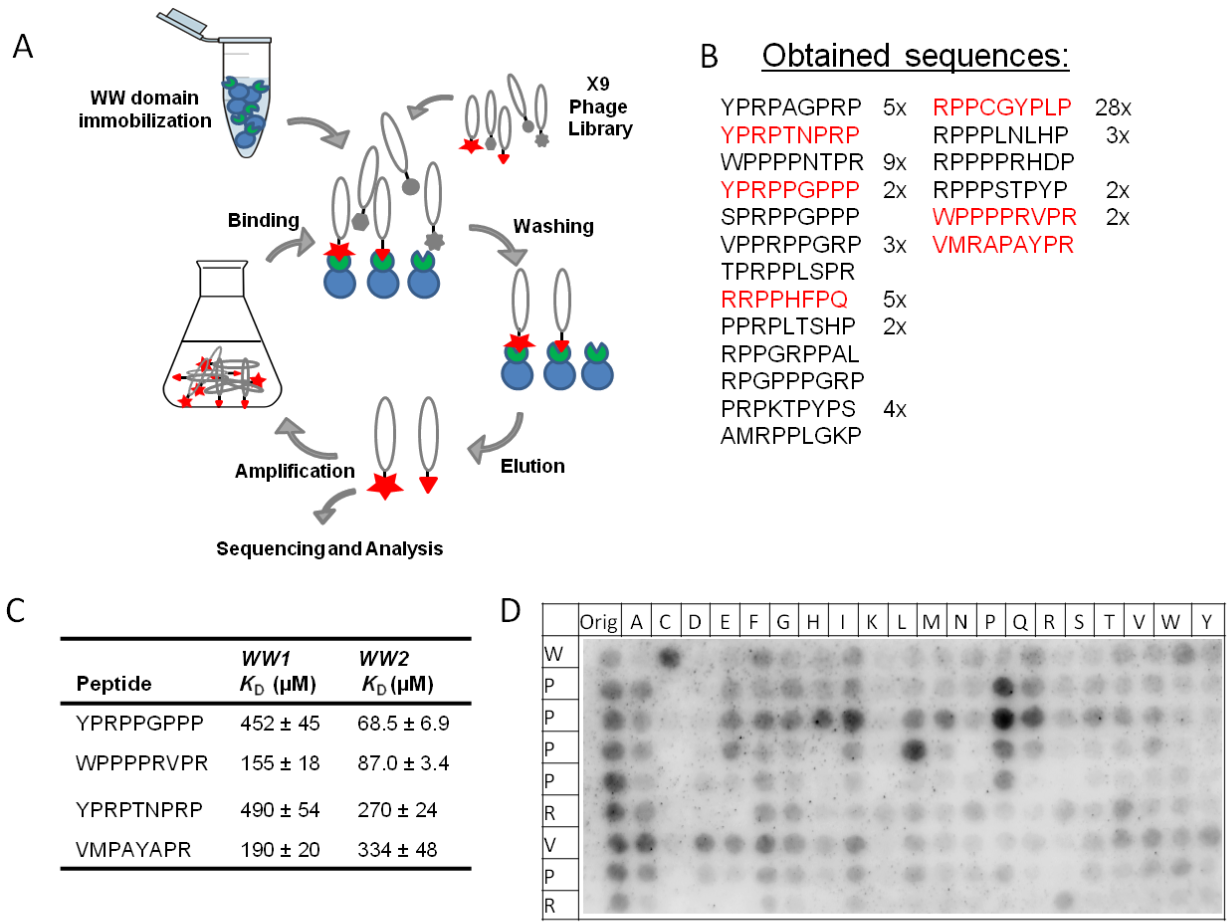
Phage display was used to find an optimized binding partner for FBP21-tWW. A) Schematic representation of the phage display workflow: FBP21-tWW is immobilized and incubated with an X9 phage library. Unbound phages are washed away, then phages expressing a binding peptide are eluted and amplified to create an enriched library, which is then subjected to another round of panning. Phages were sequenced after 3 and 4 rounds of panning. B) Sequences and frequencies of peptides obtained from eluted phages. Shown in red are the peptides which were chosen for further analysis. C) Six peptides representing different groups of binding peptides were synthesized and interactions were analyzed using ITC. Shown are the peptides for which *K*_D_ values could be determined. D) A substitution analysis showed that in the binding motif of the peptide WPPPPRVPR, P5 and R9 most strongly contribute to binding of both WW domains. Shown here is the substitution analysis for WW2, the corresponding substitution analysis for WW1 is shown in Figure S1 (Supporting Information File 1).

In order to determine an optimal peptide sequence for a FBP21-tWW ligand, we conducted a phage display experiment for each of the two WW domains. Phages used in these experiments carried a randomized X9-peptide fused to phage coat protein VIII [16]. Enrichment factors were calculated after each selection cycle, and reached a plateau after four panning rounds. Phagemids were isolated and sequenced after the third and fourth panning rounds. The sequences for WW1 and WW2 were overlapping to a large extent, showing their similarity in binding preference. This is expected from evolutionary and binding studies carried out earlier [17–18]. The sequences obtained are shown in Figure 1B. In agreement with previous findings for this group of WW domains a polyproline stretch and an arginine residue are present in most sequenced clones. Interestingly, a preference for an aromatic residue N-terminal to the polyproline stretch could also be observed. To define an optimized single monovalent binder, we analyzed the affinities for a selected set of ligands from this panel of sequences with regard to FBP21’s WW domains by ITC under the same conditions as were used for the phage display. Four of the six selected peptides showed affinities in the high micromolar range, while the peptide WPPPPRVPR showed higher affinities for both WW domains. *K*_D_ values of 155 ± 18 µM and 87.0 ± 3.4 µM were measured for WW1 and WW2, respectively (Figure 1C). The peptide which was found with the highest frequency in the phage display experiment (RPPCGYPLP), did not show any binding to either WW domain in ITC experiments, possibly reflecting the potential of the cysteine residue to form an unspecific complex with glutathione-S-transferase. Similarly, the peptide RPPPPHFPQ could not be confirmed as a binder in the ITC experiments. To further analyze which residues are essential for the interaction, we performed substitution analyses using peptide SPOT arrays (Figure 1D, Supporting Information File 1 Figure S1). The experiment gives some insight into binding specificities. The central role of proline P5 for binding of peptides is evident for both WW domains (Figure 1D, Supporting Information File 1 Figure S1). Proline fulfills two requirements. On the one hand it promotes formation of the PPII helix conformation, on the other hand, it is accommodated well into the hydrophobic groove provided by the individual WW domains [5]. At position P4 proline can be replaced by leucine, a residue well compatible with the PPII helical conformation. Arginine is preferred at position 6 or 9, highlighting the importance of a positive charge in complex formation. Interestingly, WW1 tolerates an exchange for lysine in these positions, while WW2 more strictly requires the arginine. Possibly, the hybrid resonance of the guanidinium group in the arginine is more important in the case of WW2 compared to WW1.

The peptide WPPPPRVPR was further on taken as a basis for exploring the effect of multivalent polymer display. In order not to restrict binding after coupling by shielding of the C-terminal arginine, we decided to attach a small linker to the C-terminus of the derived peptide, yielding the sequence WPPPPRVPRGSG.

### Synthesis of hPG-peptide conjugate **2**

**Scheme 1:**
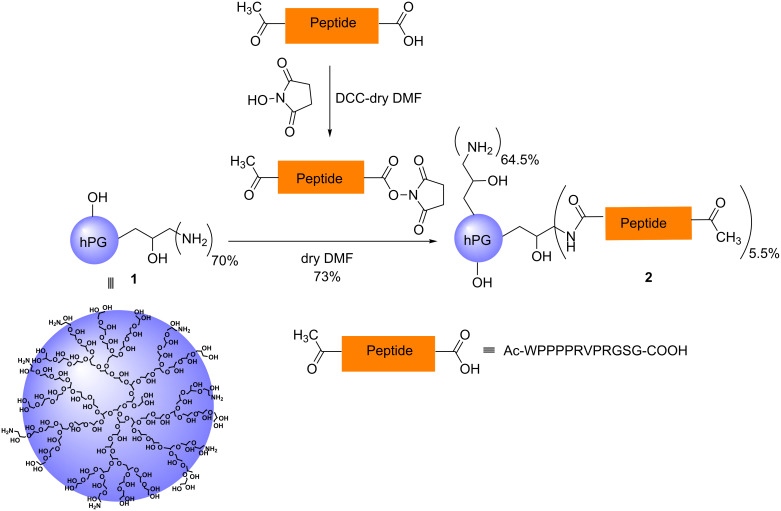
Schematic representation of the synthesis of hPG-peptide conjugate **2**.

hPG-OH (*M_n_* = 9.0 kDa, PDI = 1.86) was prepared according to the published procedure [19] (see Figures S2 and S3 in Supporting Information File 1 for GPC and MALDI–TOF–MS analysis of the hPG-OH core). Seventy percent of all hydroxy groups (≈120 OH groups) on hPG-OH were functionalized with amino groups in a three-step protocol as reported in the literature [19]. The transformation started from the mesylation of the hydroxy groups on the hPG. In the next step, the mesylated polyglycerol was converted to poly(glycerol azide). In the last step, azide functionalities (N_3_) were reduced to primary amines (-NH_2_) via Staudinger reduction to obtain the desired hPG-NH_2_ **1** (see Figure S4 in Supporting Information File 1 for GPC analysis of hPG-NH_2_ **1**). The appropriate derivative of the targeting peptide, i.e., Ac-WPPPPRVPRGSG-COOH was activated by *N*-hydroxysuccinimidyl ester formation and used for coupling with hPG-NH_2_ **1** (*M*_n_ = 7.3 kDa, PDI = 1.97) to achieve the hPG-peptide conjugate **2** in good yield as shown in Scheme 1. Only 5.5% of the amine groups on hPG-NH_2_ **1** were conjugated with peptide, as determined by ^1^H NMR analysis, keeping the rest of amine groups free on hPG-peptide conjugate **2** for its cellular penetration properties (see Figures S5 and S6 in Supporting Information File 1 for the ^1^H NMR spectra of the peptide and hPG-peptide conjugate **2**). The 5.5% peptide conjugation accounts for an average of 7.00 peptide units per polymer.

### Analysis of the inhibitory potential of hPG-peptide conjugate **2**

**Figure 2:**
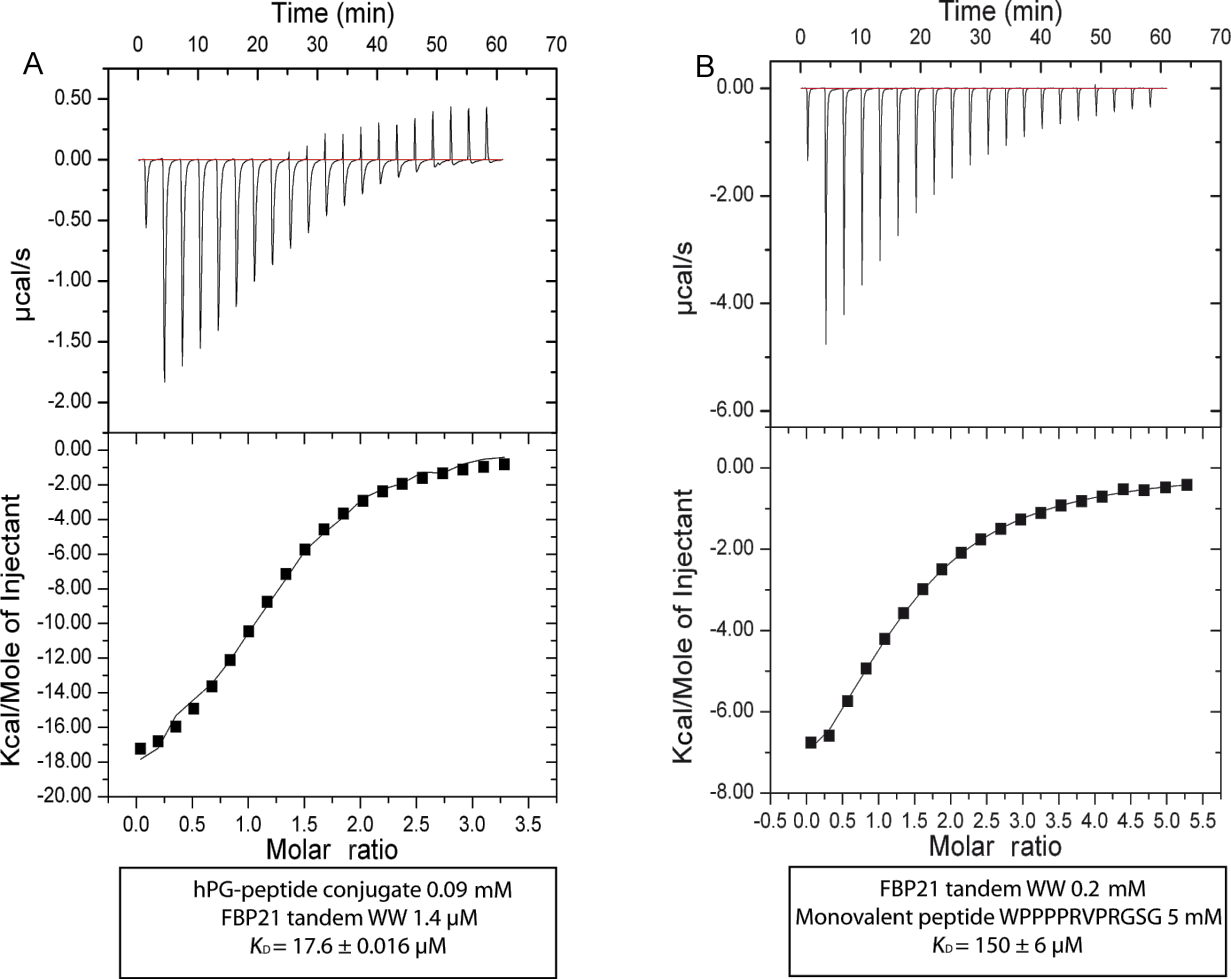
A) ITC measurement with FBP21-tWW and hPG-peptide conjugate **2**, the *K*_D_ value is 17.6 ± 0.016 μM. B) ITC measurement with FBP21-tWW and monovalent peptide shows a *K*_D_ of 150 ± 6 µM.

ITC measurements were performed to analyze the dissociation constant *K*_D_ of the interaction between FBP21-tWW and hPG-peptide conjugate **2** in PBS, pH 7.4 (Figure 2A). To compare the *K*_D_ values we also analyzed the binding between FBP21-tWW and the monovalent peptide ligand Ac-WPPPPRVPRGSG-COOH (Figure 2B). The *K*_D_ obtained with the monovalent ligand was 150 ± 6 μM in solution whereas it was 17 ± 0.016 μM for hPG-peptide conjugate **2**, demonstrating an approximately tenfold overall affinity enhancement. However, when considering that ≈7 peptides are bound per nanoparticle, the actual multivalency effect is small.

For both the monovalent ligand and the hPG-conjugate **2**, a stoichiometric factor of approximately one was derived from the fit (1.29 ± 0.0257 for monovalent ligand and tWW, 1.18 ± 0.0221 for tWW and hPG-conjugate 2) suggesting that the observed affinity is mainly conferred by a one-to-one interaction. For the hPG-conjugate this means that most peptides on the hPG particle are not engaged in the interaction with FBP21-tWW. The enthalpy of the interaction is twice as large for the hPG-peptide conjugate as for the monovalent ligand, while the entropy loss upon interaction (−19.5 cal/mol/deg for the interaction with the monovalent ligand and −45.9 cal/mol/deg for the interaction with hPG-peptide conjugate **2**) is significantly lower for the hPG-peptide conjugate, indicating that the hPG scaffold might impose certain geometric constraints on the interaction that are not present in the free peptide.

## Conclusion

In summary, we have optimized a peptide ligand for the WW domains of FBP21 and were able to enhance the binding affinity by presenting it on a multivalent dendritic polyglycerol scaffold by a factor of ten in comparison to the monovalent ligand. However, given that on an average seven peptides are presented on the nanoparticle, this overall enhancement is small and should be improved in future by varying the ligand density and size of the hPG-NH_2_.

## Supporting Information

File 1 Details on materials and methods and supplementary figures.
