# Supplementary material for: Exploring monovalent and multivalent peptides for the inhibition of FBP21-tWW
Source: Beilstein J Org Chem. 2015 May 11;11:701–6. doi: 10.3762/bjoc.11.80 (PMC4464085; doi:10.3762/bjoc.11.80)
Supplement: File 1 — Details on materials and methods and supplementary figures. [file Beilstein_J_Org_Chem-11-701-s001.pdf]

Supporting Information

for

Exploring monovalent and multivalent peptides for the inhibition of FBP21-tWW

Lisa Maria Henning[1‡], Sumati Bhatia[2‡], Miriam Bertazzon[1], Michaela Marczynke[3], Oliver Seitz[3], Rudolf Volkmer[4,5], Rainer Haag[2] and Christian Freund[1*]

Address:[1]Institute for Chemistry and Biochemistry, Protein Biochemistry Group, Thielallee 63, Freie Universität Berlin, 14195 Berlin, Germany, [2]Institute for Chemistry and Biochemistry, Freie Universität Berlin, Takustr. 3, 14195 Berlin, Germany, [3]Institute for Chemistry, Humboldt-Universität Berlin, Brook-Taylor-Str. 2, 12489 Berlin, Germany, [4]Leibniz Institut für Molekulare Pharmakologie FMP, Robert-Rössle-Str.10, 13125 Berlin, Germany and [5]Institute of Medical Immunology, Charité-Universitätsmedizin Berlin, Berlin, Germany

[*]Corresponding author

Email: Christian Freund - christian.freund@fu-berlin.de

‡ equal contributors

Details on materials and methods and supplementary figures

Experimental

Materials

All reagents and solvents were purchased from commercial suppliers and used without further purification. Reactions requiring dry or oxygen-free conditions were carried out under argon in Schlenk glassware. Peptides were synthetized by Fmoc solid phase peptide synthesis (SPPS). Dialysis tubes purchased from Spectrum Labs® with MWCO 3.5–5 kDa were used in the purification of multivalent hPG-peptide conjugate **2.**

Gel permeation chromatography (GPC)

Molecular weight distribution of hPG-OH core was determined by means of GPC coupled to a refractive index detector (RI) obtaining the complete distribution (M_n, Mp, M_w, dispersity). Measurements were carried out under highly diluted conditions (5 mg/mL) from a GPC consisting of an Agilent 1100 solvent delivery system with pump, manual injector, and an Agilent differential refractometer. Three 30 cm columns (PPS: Polymer Standards Service GmbH, Germany; Suprema 100 Å, 1000 Å, 3000 Å with 5 and 10 µm particle size) were used to separate aqueous polymer samples using water with 0.1 M $NaNO_3$ as the mobile phase at a flow rate of 1 mL/min. The columns were operated at room temperature with the RI detector at 50 °C. The calibration was performed by using certified standards pullulan standards (linear) from PSS. WinGPC Unity from PSS was used for data acquirement and interpretation.

Isothermal titration calorimetry

The His_6-FBP21-tWW, FBP21-WW1 and FBP21-WW2 were dialyzed against PBS, pH 7.4. Peptides were dissolved in PBS and the pH was adjusted to 7.4. hPG-peptide conjugate **2** was dialyzed against Millipore water and the buffer components were adjusted to PBS pH 7.4. The concentration was analyzed for all components by measuring absorbance at 280 nm using a Nanodrop2000 (Thermo Scientific). Extinction coefficients were ε = 5500 $M^{-1}cm^{-1}$ for WPPPPRVPR and ε = 32430 $M^{-1}cm^{-1}$ for FBP21-tWW. Accounting for the number of peptides on the scaffold, we calculated an extinction coefficient of ε = 38500 $M^{-1}cm^{-1}$ for the hPG-peptide conjugate **2.** ITC experiments were carried out on a Microcal ITC200 (GE Healthcare). The parameters were set to: temperature 25 °C, spacing: 120 s (for WW1 and WW2 measurements) or 180 s (for tWW measurements), stirring speed: 1000 rpm, feedback gain: high, Filter: 5 s, DP: 7 µcal/s. In ITC measurements with FBP21 WW1 and WW2, the protein in the cell was concentrated to 250 µM, the peptide in the syringe to 5 mM, injected was 1 × 1 µL, then 15 × 2.5 µL. In ITC measurements with FBP21-tWW and peptide the protein concentration in the cell was 200 µM and the peptide concentration in the syringe 4 mM. In ITC measurements with FBP21-tWW and the hPG-peptide conjugate **2**, the concentration of **2** in the cell was 90 µM and FBP21-tWW in the syringe 1.4 mM. Injected was 1 × 1 µL, then 19 × 2 µL. The data was analyzed with Origin and the Microcal ITC 200 AddOn. To obtain the stoichiometry (N) and the

association constant (K_A) of the reaction, peak areas were integrated and reaction heats were plotted against the molar ratio of titrant versus analyte. The baseline was corrected, the first point was removed. The enthalpy of salvation was substracted and the data was fitted according to the model for one binding site in the Microcal software.

Nuclear magnetic resonance (NMR)

^{1}H NMR spectra were recorded on Bruker AMX 500 (500 MHz) and Delta Joel Eclipse 700 (700 MHz) spectrometer at 25 °C and calibrated by using the peak of residual undeuterated solvent.

Mass spectrometry

The molecular weight of the hPG-OH core was confirmed by MALDI–TOF-mass using Bruker Ultraflex TOF/TOF" in the positive ion mode with a "α-cyano-4-hydroxycinnamic acid" matrix by using the linear pathway. The GPC from Agilent 1100 solvent delivery system with RI detector and Novema column was used for the determination of the molecular weight distribution of hPG-NH_2 **1**. The sample concentration was 5 mg/mL using water with 0.3 M formic acid at a flow rate of 1 mL/min.

Phage display

FBP21-WW1 and FBP21-WW2 were used as bait proteins, immobilized via their GST-tag on glutathione-sepharose beads, with GST as negative control. For each sample 40 µL 75% glutathione-sepharose slurry were used, washed and incubated with 0.3 mg of protein for 15 min at rt. The matrix was washed thoroughly before addition of approximately $2 \cdot 10^{12}$ phage particles, BSA to the final concentration 5 mg/mL and PBST to a final volume of 500 µL. The samples were incubated overnight at 4 °C, washed thoroughly and eluted by the addition of 350 µL 0.1 M glycine (pH 2.2) for 15 min. For calculating enrichment and sequencing phagemids, a dilution series of phages was prepared and used for infection of *E. coli*. To amplify the eluted phages, they were used to infect an *E. Coli XL1-Blue* culture. The culture was grown at 37 °C, 180 rpm until an OD600 of 0.5 was reached. The bacteria were co-infected with 10^{11}–10^{12} helper phage

particles and grown overnight at 30 °C, 250 rpm. The bacteria were pelleted; Phages were precipitated from the supernatant using 20% PEG, 2.5 M NaCl. The precipitated phages were pelleted and washed before resuspension in PBS/30% glycerol.

Protein expression and purification

FBP21-WW1 and FBP21-WW2 were expressed as a GST-tagged variant from pgex4T1 in *E. coli* BL21 DE3, FBP21-tWW was expressed from a pET28a-His_6-FBP21-tWW plasmid in *E. coli* BL21 DE3 as described previously [5]. Proteins were purified by the corresponding affinity matrices and applied to a HiLoad S75 Superdex 16/60 column in 1 × PBS, pH 7.4. Proteins were concentrated by Centrifugal Filter Units (regenerated cellulose, 3000 MWCO, Millipore).

Substitution analysis

SPOT analyses were performed as described [1]. Membranes were blocked with 5% BSA in PBS, washed and incubated with GST, GST-WW1 or GST-WW2 at a concentration of 7.5 µg/mL (GST) or 10 µg/mL (GST-WW1, GST-WW2) respectively overnight at 4 °C. After washing, the spot arrays were incubated with a primary anti-GST antibody (Santa Cruz, GST-Z5, sc-459, 1:1000) for one hour at room temperature in blocking buffer, washed again and incubated for one hour with a secondary anti-rabbit antibody (goat anti-rabbit, Santa Cruz, sc-2004, 1:10000) at room temperature in blocking buffer. The peptide spot arrays were developed with HRP juice purchased from pjk GmbH on an Intas Advanced Fluorescence and ECL imager.

Synthesis of hPG-peptide conjugate 2

The peptide carboxylic acid, i.e., Ac-WPPPPRVPRGSG-COOH (0.0067 mmol, 9 mg), was dissolved in 2 mL dry DMF and cooled down to 0 °C using an ice bath under argon atmosphere. To the cooled solution, *N*-hydroxysuccinimide (0.013 mmol, 1.54 mg) and DCC (0.013 mmol, 2.76 mg) were added. The ice bath was removed and the reaction mixture was allowed to stir at room temperature overnight. The solution of hPG-NH_2 **1** (10 mg, 0.0067 mmol of NH_2 to be functionalized) in methanol was dried carefully under high vacuum and diluted with 1 mL of dry DMF. The dry DMF was evaporated again to get rid of traces of methanol. The residue was

diluted with dry DMF (1 mL). The already stirring solution of succinimidyl-peptide ester in DMF was added to the solution of hPG-NH_2 **1** in dry DMF under argon atmosphere. The reaction mixture was stirred at room temperature overnight and transferred directly to the dialysis tube with 3.5–5 kDa MWCO and dialyzed with methanol for 2 days followed by dialysis in millipore water for 2 days. After the completion of dialysis, a small volume of the aqueous solution was lyophilized to obtain the total mass of the product obtained after dialysis. The final pure product was obtained in 73% yield affording a total mass of 14.6 mg. The degree of peptide conjugation obtained by ^{1}H NMR (700 MHz) was 5.5% which accounts for 7 peptide units per hPG nanoparticle. The average molecular weight of the hPG-peptide conjugate **2**, based on the average molecular weight of the hPG-OH core and degree of peptide conjugation as obtained by ^{1}H NMR, was ~18 kDa. ^{1}H NMR (500 MHz, D_2O): δ = 0.94–0.99 [m, 6H, $(CH_3)_2CH$ of Val residue], 1.30–2.08 (m, $NHCOCH_3$, 2×$CH_2CH_2CH_2NH$ of Arg residue, 5× CH_2CH_2 of Pro residue), 2.29–2.83 (m, CH_2NH_2 of hPG backbone, $(CH_3)_2CH$ of Val residue), 3.06–4.14 (m, 5×CH_2 of Pro residue, hPG backbone, $NHCOCHCH_2$ of Trp residue, 2×$CH_2CH_2CH_2NH$ of Arg residue, 2×CH_2 of Gly residue, CH_2OH of Ser residue), 4.34–4.64 (m, 5×NC*H* of Pro residue, $CHCH_2OH$ of Ser residue, $CHCH(CH_3)_2$ of Val residue), 7.27–7.32 (m, 3H, ArH of Trp residue), 7.51–7.98 (m, 1H, ArH of Trp residue), 7.71–7.74 (m, 1H, ArH of Trp residue) ppm.

References:

1. Kofler, M; Heuer, K.; Zech, T.; Freund, C. *J Biol Chem.* **2004**, 279, 28292-28297.

Supplementary Figures.

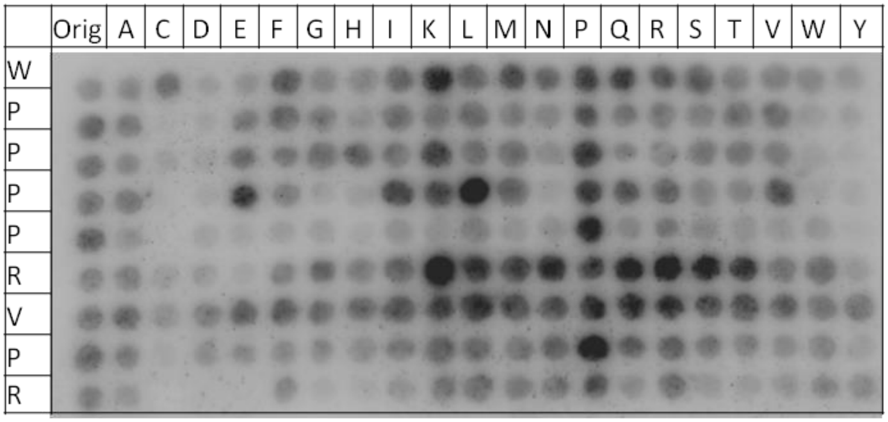

Figure S1: Substitution analysis of the peptide sequence WPPPPRVPR with FBP21 WW1. WW1 shows in general higher binding affinities to proline-rich peptides, but like for WW2, P5 and a positive charge are required for the interaction.

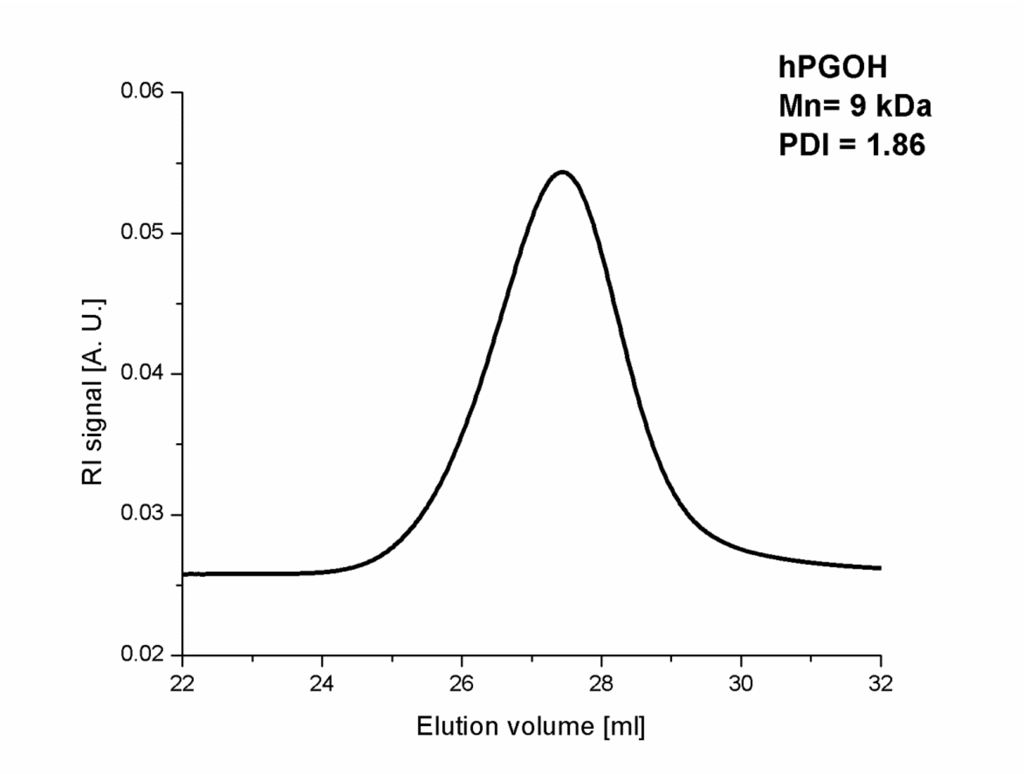

Figure S2: Analytical GPC data of hPG-OH.

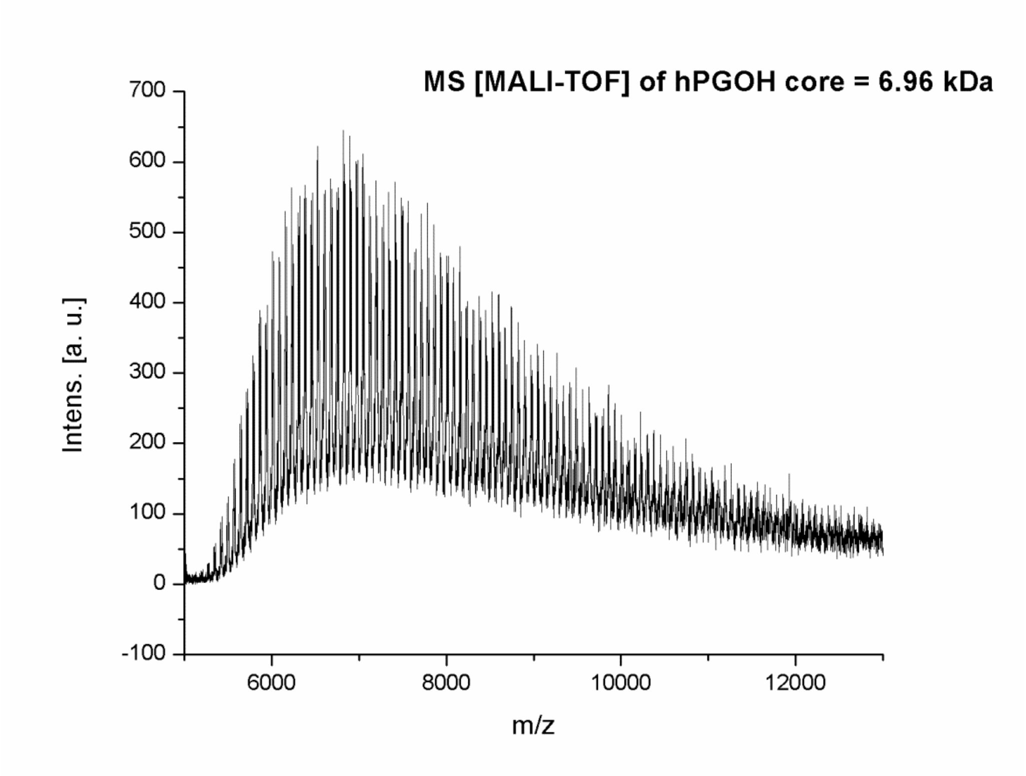

Figure S3: MS (MALDI–TOF) data of hPG-OH.

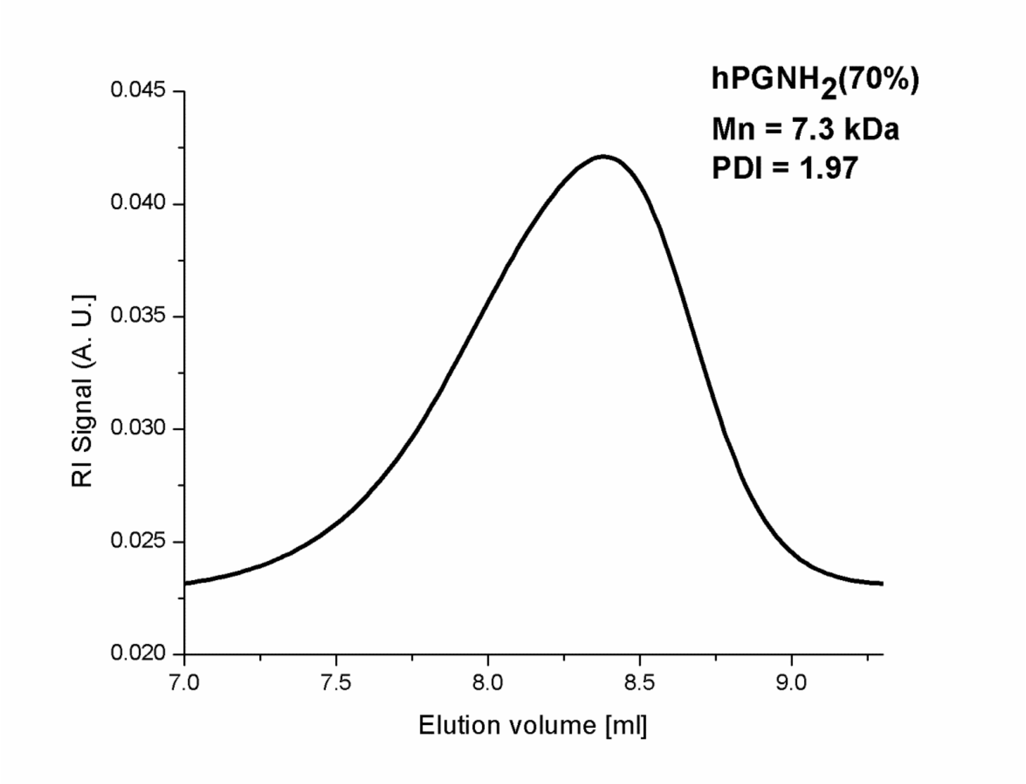

Figure S4: Analytical GPC data of hPG-NH_2 **1**.

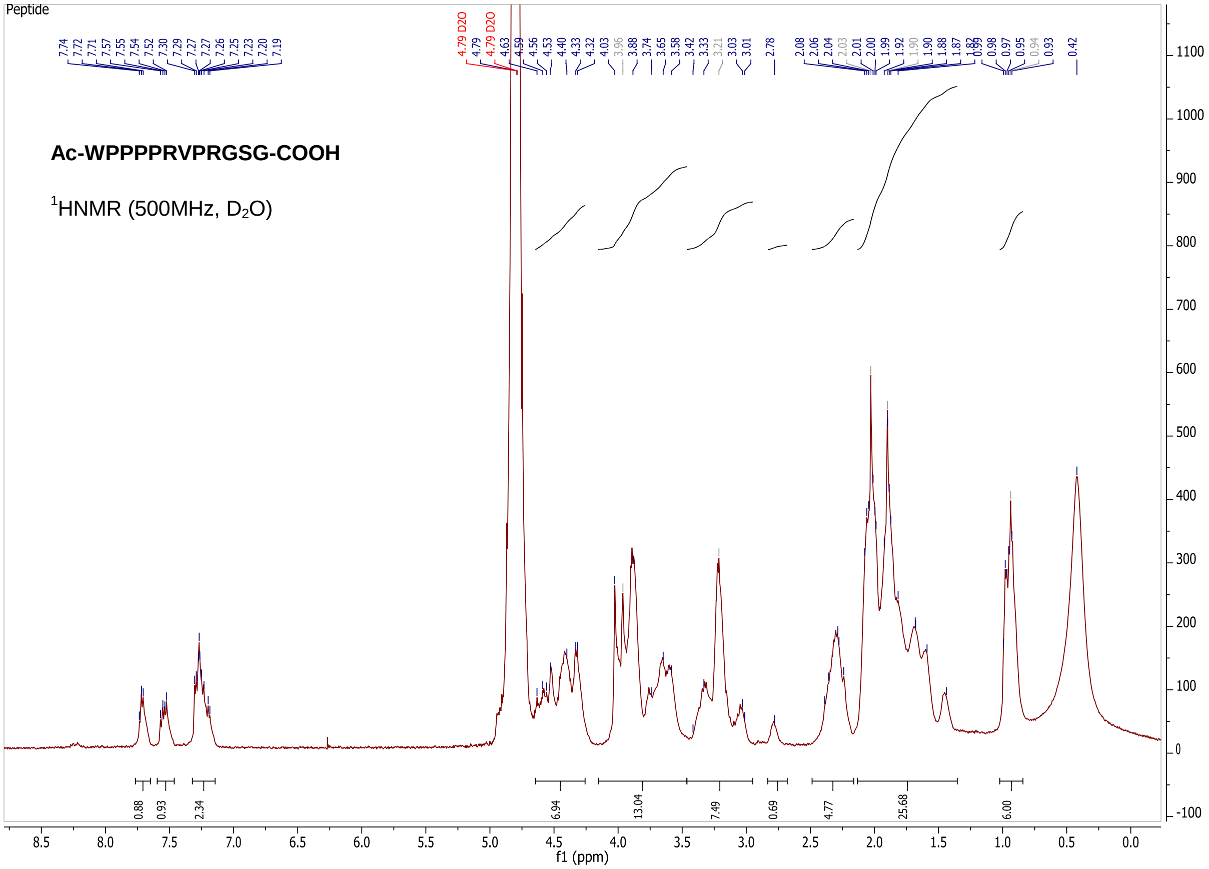

Figure S5: ^{1}H NMR (500 MHz, D_2O) of Ac-WPPPPRVPRGSG-COOH.

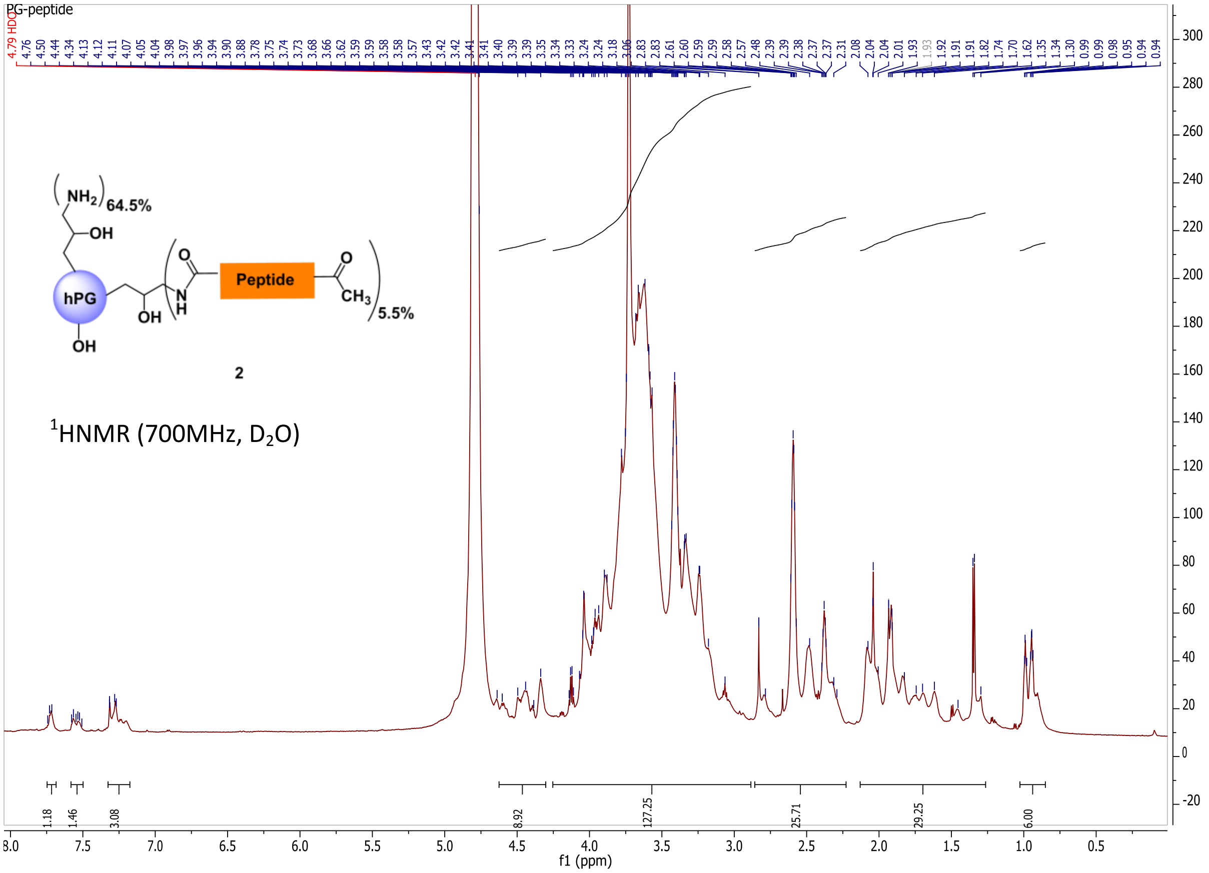

Figure S6: ^{1}H NMR (700 MHz, D_2O) of hPG-peptide conjugate **2**.
